# Definitions, Biology, and Current Therapeutic Landscape of Myelodysplastic/Myeloproliferative Neoplasms

**DOI:** 10.3390/cancers15153815

**Published:** 2023-07-27

**Authors:** Margo B. Gerke, Ilias Christodoulou, Theodoros Karantanos

**Affiliations:** 1 School of Medicine, Emory University, Atlanta, GA 30322, USA; mgerke@emory.edu; 2 Department of Medicine, University of Pittsburgh, Pittsburgh, PA 15213, USA; christodouloui@upmc.edu; 3 Department of Oncology, Johns Hopkins University, Baltimore, MD 21218, USA

**Keywords:** myelodysplastic/myeloproliferative neoplasms, MDS/MPN overlap syndromes, CMML, aCML, MDS/MPN-T-SF3B1, MDS/MPN-RS-T-NOS, MDS/MPN-NOS

## Abstract

**Simple Summary:**

Myelodysplastic/myeloproliferative (MDS/MPN) neoplasms are blood disorders characterized by abnormal cell growth and development. These disorders encompass various subtypes, including chronic myelomonocytic leukemia, atypical chronic myeloid leukemia, and others. They are caused by genetic changes in different cell components and have distinct clinical features. Current treatment options mostly involve drugs that control the disease, including hypomethylating agents, ruxolitinib, lenalidomide, and venetoclax, but do not offer a cure. However, allogeneic bone marrow transplantation has the potential to cure these disorders. Several factors, such as overall health, spleen enlargement, and genetic alterations, can influence the outcome of transplantation. Future research is crucial to improving treatment approaches and patient outcomes for MDS/MPN neoplasms. This review provides an overview of the diagnosis, biology, and current and upcoming treatments, including bone marrow transplantation, for these complex blood disorders. This review will shed light on the complexities of MDS/MPN neoplasms and will inform future research for improved therapeutic strategies and patient care in the future.

**Abstract:**

Myelodysplastic/myeloproliferative neoplasms (MDS/MPN) are hematological disorders characterized by both proliferative and dysplastic features. According to the 2022 International Consensus Classification (ICC), MDS/MPN consists of clonal monocytosis of undetermined significance (CMUS), chronic myelomonocytic leukemia (CMML), atypical chronic myeloid leukemia (aCML), MDS/MPN with SF3B1 mutation (MDS/MPN-T-SF3B1), MDS/MPN with ring sideroblasts and thrombocytosis not otherwise specified (MDS/MPN-RS-T-NOS), and MDS/MPN-NOS. These disorders exhibit a diverse range of genetic alterations involving various transcription factors (e.g., *RUNX1*), signaling molecules (e.g., *NRAS*, *JAK2*), splicing factors (e.g., *SF3B*, *SRSF2*), and epigenetic regulators (e.g., *TET2*, *ASXL1*, *DNMT3A*), as well as specific cytogenetic abnormalities (e.g., 8 trisomies, 7 deletions/monosomies). Clinical studies exploring therapeutic options for higher-risk MDS/MPN overlap syndromes mostly involve hypomethylating agents, but other treatments such as lenalidomide and targeted agents such as JAK inhibitors and inhibitors targeting PARP, histone deacetylases, and the Ras pathway are under investigation. While these treatment modalities can provide partial disease control, allogeneic bone marrow transplantation (allo-BMT) is the only potentially curative option for patients. Important prognostic factors correlating with outcomes after allo-BMT include comorbidities, splenomegaly, karyotype alterations, and the bone marrow blasts percentage at the time of transplantation. Future research is imperative to optimizing therapeutic strategies and enhancing patient outcomes in MDS/MPN neoplasms. In this review, we summarize MDS/MPN diagnostic criteria, biology, and current and future treatment options, including bone marrow transplantation.

## 1. Introduction

Myelodysplastic/myeloproliferative neoplasms (MDS/MPN) are a distinct group of hematological disorders characterized by overlapping features of myelodysplastic syndromes (MDS) and myeloproliferative neoplasms (MPN). Both elements are required to diagnose MDS/MPN, with the identification of the hyperproliferation of hematopoietic cells in conjunction with bone marrow dysplasia and ineffective hematopoiesis, which occasionally leads to cytopenia. In instances where cytopenia is not present, morphological analysis of the bone marrow may show dysplastic changes in one or more lineages, fulfilling the criteria for MDS/MPN diagnosis.

In recent years, the classification and understanding of MDS/MPN overlap syndromes have evolved, shedding light on their complex biology and genetic abnormalities. Traditionally, the classification of MDS/MPN was based on the fourth edition of the *WHO Classification of Tumours of Haematopoietic and Lymphoid Tissues* (WHO4), first released in 2008 and updated in 2016, which divided MDS/MPN into four subdivisions: chronic myelomonocytic leukemia (CMML), atypical chronic myeloid leukemia (aCML), myelodysplastic/myeloproliferative neoplasm with ringed sideroblasts and thrombocytosis (MDS/MPN-RS-T), MDS/MPN-unclassifiable (MDS/MPN-U), and Juvenile myelomonocytic leukemia (JMML) [1]. In 2022, the most recent edition (5th-WHO5) was released, which still included CMML but introduced changes: JMML was removed, aCML was renamed to MDS/MPN with neutrophilia (mainly to avoid confusion with CML), MDS/MPN-RS-T was renamed as MDS/MPN with thrombocytosis and SF3B1 mutation (MDS/MPN-T-SF3B1), and MDS/MPN-unclassifiable was termed as MDS/MPN not otherwise specified (MDS/MPN-NOS) [2]. The same year, the International Consensus Classification (ICC) of Myeloid Neoplasms and Acute Leukemias divided MDS/MPN into seven subdivisions: CMML, clonal monocytosis of undermined significance (CMUS), aCML, MDS/MPN-T-SF3B1, MDS/MPN-RS-T-not otherwise specified (MDS/MPN-RS-T-NOS), and MDS/MPN-NOS [3]. Given the absence of identifiable dysplastic features, JMML was excluded from the most recent MDS/MPN classifications and was included in MPN on the WHO5 and in pediatric/germline mutation-associated disorders on the ICC, highlighting the evolving nature of classification systems.

The frequency of MDS/MPN overlap syndromes varies, with CMML accounting for most cases, with an incidence ratio of 0.6 per 100,000 patients, followed by aCML (0.06 per 100,000 patients) [4]. All MDS/MPN can potentially progress into AML (around 15–40% in 3–5 years) [5]. Understanding these entities’ clinical presentation and biology is vital for proper diagnosis and management. In this review, we will list the diagnostic criteria of MDS/MPN based on the 2022 ICC and discuss the biology of these diseases—particularly, their genetic profile. Moreover, we will highlight the current therapeutic landscape of these diseases and outline outcomes and considerations for the allogeneic bone marrow transplantation (allo-BMT), the only potentially curative option for people with MDS/MPN.

## 2. Definitions

### 2.1. Chronic Myelomonocytic Leukemia

CMML is defined as monocytosis (absolute monocytes ≥ 0.5 × 10^9^/L and ≥10% of the WBC) with cytopenia and the presence of <20% of blasts in the peripheral blood (PB) and bone marrow (BM) [3]. A clonal population with abnormal cytogenetics or myeloid neoplasm-associated mutation is needed for diagnosis unless monocytes > 1 × 10^9^/L [3,6]. BM typically demonstrates hypercellularity due to the proliferation of myeloid lineage without the pathological features of AML, MPN, or other monocytosis-associated conditions [3]. Moreover, BCR-ABL1 translocation or other genetic abnormalities of myeloid/lymphoid neoplasms with eosinophilia and tyrosine kinase gene fusions should not be detected [3]. Of note, two subgroups of CMML have been defined based on blast percentage: CMML-1 with <5% blasts in PB and <10% in BM, and CMML-2 with 5–19% blasts in PB and 10–19% in BM [3]. Finally, the amount of total WBC classifies CMML into two additional groups: the myeloproliferative subtype (MP-CMML) with WBC of >13 × 10^9^/L and the myelodysplastic group (MD-CMML) with lower WBC counts [3].

### 2.2. Atypical Chronic Myeloid Leukemia

ACML is defined as leukocytosis 13 × 10^9^/L due to an increased number of neutrophils and their precursors, cytopenia, and blasts less than 20% of the composition of cells in PB and BM. Dysgranulopoiesis is present in PB, including hyposegmented and/or hypersegmented neutrophils and hypercellular BM with granulocytic proliferation and dysplasia [3]. Monocytes and eosinophils constitute <10% of PB leukocytes each [3]. Like CMML, BCR-ABL1 translocation or other genetic abnormalities of myeloid/lymphoid neoplasms with eosinophilia and tyrosine kinase gene fusions should not be detected [3].

### 2.3. Clonal Monocytosis of Undetermined Significance

CMUS is a premalignant precursor to CMML defined by monocytosis (absolute monocytes ≥ 0.5 × 10^9^/L and ≥10% of the WBC) with the presence or absence of cytopenia, in the presence of myeloid neoplasm-associated mutation [3]. No morphological findings of CMML, dysplasia, or blasts should be identified in bone marrow, and no other criteria for hematologic neoplasm should be met, while other causes of reactive monocytosis should be excluded [3]. In the presence of cytopenia, CMUS can be re-named as Clonal Cytopenia with Monocytosis of Undetermined Significance (CCMUS) [3].

### 2.4. Myelodysplastic/Myeloproliferative Neoplasm with Thrombocytosis and SF3B1 Mutation

MDS/MPN with thrombocytosis and *SF3B1* mutation (MDS/MPN-T-*SF3B1*) is defined as thrombocytosis (platelets > 450,000 × 10^9^/L) with anemia with minimal blasts (<1% in PB and <5% in BM) [3]. The *S3FB1* mutation is necessary for diagnosis, with or without other myeloid neoplasm-associated mutations or abnormal cytogenetics, but always without BCR-ABL1 translocation or other genetic abnormalities of myeloid/lymphoid neoplasms with eosinophilia and tyrosine kinase gene fusion [3]. MDS, MPN, or other MDS/MPN should be excluded, along with a history of growth factors which could be responsible for myeloproliferative features or cytotoxic therapy responsible for myelodysplasia [3].

### 2.5. Myelodysplastic/Myeloproliferative Neoplasm with Ring Sideroblasts and Thrombocytosis, Not Otherwise Specified

MDS/MPN-RS-T, NOS is defined as thrombocytosis (platelets > 450,000 × 10^9^/L) with minimal blasts (<1% in PB and <5% in BM) and anemia with erythroid-lineage dysplasia and >15% ring sideroblasts [3]. A clonal population with abnormal cytogenetics or somatic mutation(s) but no *S3FB1* mutation, BCR-ABL1 translocation, or other genetic abnormalities of myeloid/lymphoid neoplasms with eosinophilia and tyrosine kinase gene fusion should be identified. The presence of other MDS, MPN, or MDS/MPN needs to be excluded [3].

### 2.6. Myelodysplastic/Myeloproliferative Neoplasm, Not Otherwise Specified

MDS/MPN-NOS entails cytopenias, thrombocytosis, or leukocytosis (as above) and blasts in PB and BM < 20% [3]. Clonality, as displayed by clonal cytogenetic and/or somatic mutation(s), must be present, but not the BCR-ABL1 translocation or other genetic abnormalities of myeloid/lymphoid neoplasms with eosinophilia and tyrosine kinase gene fusion [3]. The absence of any other MDS/MPN, MDS, MPN, or history of prior cytotoxic or growth factor therapy is required [3].

## 3. Biology

### 3.1. Genetic Mutations

The genomic profile of patients with MDS/MPN defines the disease biology and strongly affects patients’ outcomes [7]. Palomo et al. demonstrated that *TET2* and *SRSF2* mutations are the most common founder mutations in CMML, while *ASXL1*/*SETBP1* mutations co-occur frequently in aCML [7]. The authors demonstrated that MDS/MPN-NOS (formerly, MDS/MPN-unclassifiable) is the most heterogeneous group with the molecular profile defining disease progression and outcomes [7]. Finally, it was highlighted that TP53 mutation defines a separate phenotype characterized by dismal outcomes [7]. Our group showed that men with MDS/MPN neoplasms have a higher number of somatic mutations and a greater number of high-risk mutations (*ASXL1*, *EZH2*, *RUNX1*, *SETBP1*, *NRAS*, *STAG2*), which were associated with a higher risk of AML transformation and worse survival [8].

Somatic genetic mutations are present in over 90% of patients with CMML, can aid in the confirmation of diagnosis, are predictive of the disease course, and present opportunities for developing novel therapeutics [3,9]. Some of the most frequently mutated genes in CMML are implicated in cellular processes, including epigenetic control, such as *TET2* and *ASXL1*; RNA splicing, including *SRSF2*; cell signaling, such as *CBL* and *NRAS*; and transcription and nucleosome assembly, including *RUNX1* and *SETBP1* [9,10,11,12,13,14,15,16,17]. *SRSF2*, *TET2*, and *ASXL1* are reported to be the most frequently mutated of these genes, present in approximately 40% of patients with CMML [7,18]. *TET2* is thought to be the initial driver mutation responsible for monocytosis, but it has not been shown to impact OS or LFS in CMML [9,10,19]. The accumulation of additional mutations such as *ASXL1*, along with *DNMT3A*, *RUNX1*, *SETBP1*, *NRAS*, *KRAS*, *CBL*, and *JAK2*, is associated with increased proliferation, dysplasia, and progression to AML [7,9,20,21,22]. Nonsense/frameshift *ASXL1* mutations are the only mutations independently and consistently associated with poor prognosis [9]. ASXL1 mutations have been incorporated as a predictive component in several prognostic CMML scoring systems, including the Groupe Francophone des Myelodysplasias (GFM) Model, Mayo Molecular Model, and Spanish CMML specific cytogenetic risk stratification model (CPSS) [9,10,18,23,24,25]. In CMUS, these listed myeloid mutations and their frequency are associated with an increased risk of disease progression to CMML [3,26].

In aCML, the absence of MPN-associated driver mutations, such as *JAK2*, *CALR*, and *MPL*, and the presence of *SETBP1* and *ASXL1* mutations can provide additional support for aCML diagnosis, according to ICC guidelines [3,27]. Palomo et al. found that approximately 90% of 71 aCML patients have an *ASXL1* mutation, found in the ancestral clone in 79% of cases [7]. Patnaik et al. analyzed 25 aCML patients reporting *ASXL1* (28%), *TET2* (16%), *NRAS* (16%), *SETBP1* (12%), and *RUNX1* (12%) as the most prevalent mutations [28]. In this study, *TET2*, *NRAS*, and *PTPN11* mutations, along with the presence of more than three mutations, were found to adversely impact survival in univariate analysis, while ASXL1, SETBP1, and ETNK1 were not found to impact prognosis [28]. In contrast with genetic prognostic studies in CMML, *TET2* was the only mutation that retained association with a worse prognosis outcome in multivariate analysis [28].

The *SF3B1* spliceosome mutation is commonly a founder mutation in MDS/MPN-RS-T, and thus, the 2022 ICC recognized the mutation as a requirement in the diagnosis of MDS/MPN-T-SF3B1, as mentioned [3,29]. Other spliceosome mutations, including *U2AF1* and *SRSF2*, are frequent founder mutations in patients without *SF3B1* mutation in MDS/MPN-RS-T NOS [29]. The *JAK2V617F* mutation is reported in 58% of MDS/MPN-RS-T patients and associated with myeloproliferative features [30]. Additional mutations in genes implicated in kinase signaling pathways, such as *NF1*, *SETBP1*, *CBL*, *FLT3*, and *MPL*, have also been frequently reported in MDS/MPN-RS-T neoplasms [30]. Due to the high frequency of *JAK2V617F* mutation, the IWG suggests that its presence supports the diagnosis of MDS/MPN-RS-T [3].

### 3.2. Chromosomal Abnormalities

Cytogenic abnormalities are present in approximately 30% of all patients with CMML [3,9,26]. An analysis of 414 CMML patients found cytogenetics to be an independent prognostic factor for OS and AML transformation (*p* = 0.001) [31]. In this analysis, the highest risk cytogenetics included the presence of trisomy 8, abnormalities of chromosome 7, or complex karyotype; an intermediate risk constituted all other chromosome abnormalities; and a low risk included a normal karyotype or the loss of the Y chromosome [31]. Stratification by these cytogenetic abnormalities divided patients into the 5-year OS of 4%, 26%, and 35% (*p* < 0.001), respectively [31]. The Mayo Clinic–French consortium studied 409 patients with CMML, also finding that 30% of patients had chromosomal abnormalities [32]. High-risk (complex and monosome karyotypes), intermediate (abnormalities not included in the high or low groups), and low-risk (normal and sole Y- or 3q) displayed median survivals of 3, 20, and 41 months, respectively [32]. *ASXL1* mutations were detected in 37% of patients with abnormal karyotypes, while *SP3B1* mutations were detected in 46% of patients with normal karyotypes [32].

In an analysis of 367 patients with different subsets of MDS/MPN, aCML and MDS/MPN-NOS were associated with the highest genomic instability: 42% and 47% of patients had chromosomal abnormalities, respectively [7]. Palomo et al. reported the most common cytogenetic abnormalities in aCML and MDS/MPN-NOS to be trisomy 8, −7/del7q, and -Y [7]. Patnaik et al. found trisomy 8, trisomy 9, and trisomy 21 to be the most common karyotype abnormalities in an analysis of 25 patients with aCML [28]. An analysis of 71 patients with MDS/MPN-RS-T demonstrated karyotype abnormalities in only 10% of patients, the most common of which being trisomy 8 (4%) and the loss of chromosome Y (4%) [33].

### 3.3. Current Therapeutic Strategies in MDS/MPN

#### 3.3.1. Hypomethylating Agents

To date, the mainstay of first-line chemotherapy for MDS/MPN includes hypomethylating agents (HMA). HMA, including 5-azacitidine (AZA) and decitabine (DAC), decrease oncogenesis-related DNA methylation by irreversibly binding to DNA methyltransferase and can also cause direct DNA damage [34,35,36,37]. HMA remains the only FDA-approved chemotherapy agent for CMML, with approval primarily gained by including patients with CMML in MDS-focused clinical trials [9,38,39,40]. In a hallmark study, Silverman et al. reported outcomes for 14 CMML patients in a randomized trial of AZA in patients with MDS, reporting that 8% of patients had a complete response (CR), 15% had a partial response (PR), and 38% demonstrated improvement within the overall study population [38]. Fenaux et al. included 11 CMML patients in a randomized phase III trial of high-risk MDS patients, reporting a median overall survival (OS) of 24.5 months with AZA treatment compared to 15 months for conventional care [39]. The overall response rate (ORR) was 29% with AZA treatment, compared to 12% in conventional care regimes [39]. Kantarjian et al. reported a 17% response rate to DAC in MDS patients compared to 0% in the supportive care group, including 14 patients with CMML among 170 total patients [40]. These studies supporting FDA approval primarily focused on MD-CMML subset patients.

Since then, clinical trials of HMA in CMML-specific populations have been performed. A meta-analysis of fourteen pooled studies, including 600 patients with CMML, identified an estimated ORR that was very similar between the DAC and AZA groups, but with CR rates slightly higher in patients treated with DAC (23% in DAC vs. 10% in AZA) [41]. Drummond et al. conducted a multicenter phase II study of AZA in 32 CMML patients, identifying an ORR of 20% with a CR of 7% [42]. No OS difference between responders and non-responders was reported [42]. Eight patients had available bone marrow (BM) samples; seven had DNA methylation reduction after six months of treatment, indicating biological activity in response to HMA [42]. A study of 121 CMML patients receiving HMA showed an ORR of 56% by the IWG MDS/MPN response criteria, with CR rates less than 20% [43]. However, 29% of CMML patients who achieved CR progressed to AML, with a median OS of 8 months, highlighting the minimal impact of AZA on CMML progression [43]. A phase II trial of DAC in 43 CMML patients yielded an ORR of 47.6% and a median OS of 17 months [44]. CMML patients with dysplastic disease were more likely to respond to DAC than patients with proliferative disease [44]. Other retrospective studies have demonstrated between 7 and45% CR rates and OR response rates between 17 and 75% in patients with MDS/MPN treated with HMA [9,42,44,45,46,47].

HMA are also used as a first-line treatment in non-CMML MDS/MPN, with their rationale for use drawn mainly from CMML studies. Case reports have explored the use of DAC in aCML, with pooled data identifying a complete hematologic remission (CHR) in seven of eight patients, with two patients being bridged to transplant [48,49,50,51,52]. Despite the small sample size, HMA are recommended in aCML as bridge-to-transplant or in cases of ineligibility for transplant or clinical trial enrollment [48]. In the analysis of 135 patients with MDS/MPN-NOS, 27 patients received ≥six cycles of HMA; 1 patient achieved a CR, 2 patients achieved a PR, 2 patients achieved a marrow response (MR), and 1 patient achieved a complete cytogenetic remission, together leading to an ORR of 19% (n = 5) [53]. Of these five respondents, two patients eventually progressed to AML [53]. In a retrospective analysis of 52 MDS/MPN-RS-T patients, 12 had received HMA therapy [54]. The ORR was 25%, with a median duration of response of 7 months, with one CR, two patients with hematological improvement, and three patients proceeding to allo-HSCT after treatment failure [54]. Melody et al. retrospectively found, in 33 MDS/MPN-RS-T patients, that AZA was associated with hematological improvement in 15% of patients [55]. In a retrospective study of 167 patients with MDS/MPN-RS-T, 45 patients (27%) were treated with HMA, with an ORR of 24% in an average treatment duration of 6 months [56].

HMA must be given IV due to cytidine deaminase, an enzyme that breaks down DAC and AZA in the small intestine and liver, reducing HMA effectiveness when taken orally. This poses challenges for patients limited by transportation and can reduce quality of life due to the excess time spent in hospitalization receiving care [57]. Cedazuridine inhibits cytidine deaminase, preventing DAC degradation and allowing for efficient delivery when co-taken orally. Savona et al. investigated the combination of DAC with cedazuridine in a phase I trial including six CMML patients, identifying similar pharmacokinetics with IV DAC and a similar safety profile [58]. Garcia-Manero et al. reported in a phase II study including 17 CMML patients that oral decitabine equivalent to standard decitabine IV 20 mg/m^2^ had similar DNA methylation, efficacy, and safety in the initial two cycles [59]. A phase III study demonstrated the similar safety, pharmacokinetics, and efficacy of oral DAC/cedazuridine (35/100 mg) with five days of IV decitabine 20 mg/m^2^ [60].

Other enhancements to HMA therapy include guadecitabine, a next-generation hypomethylating agent with prolonged metabolic activity. Guadecitabine has been tolerated in a study of high-risk myelodysplastic syndromes including 22 CMML patients [61]. The ORR for patients with CMML was 45% (10 out of 22) [61]. Phase III investigation is ongoing (NCT02907359).

Overall, marginal response rates, the development of HMA resistance, and a lack of alterations in the disease course warrant the investigation of novel agents in MDS/MPN [62]. Similarly, the discovery of markers predicting the response to HMA is lacking. Our group showed that the presence of mutations in *SETBP1*, *RUNX1*, or *EZH2* genes is associated with a worse response to HMA and inferior OS in a cohort of patients with MDS/MPN treated with AZA or DAC [24]. Duchmann et al. found that *ASXL1* gene mutations predicted lower ORR to HMA (OR = 0.85), while *TET2*^mut^/*ASXL1*^wt^ was found to be predictive of a higher CR rate (odds ratio (OR) = 1.18) and a better OS in multivariate analysis [63]. The *RUNX1* mutation was similarly associated with a worse OS, along with mutation in the *CBL* gene and higher WBCs [63,64]. Further analysis is required to identify subsets of patients with MDS/MPN with a high probability of a response to HMA.

#### 3.3.2. Ruxolitinib

Ruxolitinib is a JAK1/2 inhibitor with approved use in polycythemia vera, myelofibrosis, and acute and chronic graft-versus-host disease. A preclinical study of CMML found that the pro-neoplastic JAK1/2 pathway can be induced by the granulocyte-macrophage-colony-stimulating factor (GM-CSF) in CMML primary samples and can be successfully targeted by JAK inhibitors, including ruxolitinib [65]. Several clinical studies have explored the use of ruxolitinib in MDS/MPN. Padron et al. reported in a phase I clinical trial of CMML patients that 20 mg of ruxolitinib twice daily led to an ORR of 35% (defined by a greater than 50% spleen reduction or MDS IWG criteria) and no hematological toxicities and was associated with patient-reported symptom improvement [66]. In contrast, Abaza et al. did not observe a clinical response in a phase I trial of ruxolitinib in CMML patients [67]. These outcome differences may stem from the heterogeneity of CMML; 70% of patients in the Padron et al. study had MP-CMML, while only 16% of patients had MP-CMML in Abaza et al. [66,67]. A combined phase I/II clinical trial reported an ORR of 38% using MDS/MPN IWG criteria and a 43% spleen reduction response in CMML patients receiving 20 mg of ruxolitinib twice daily [68]. Patient-derived murine xenografts supported this finding in a combined clinical/preclinical study [68].

The identification of mutations of the colony-stimulating factor 3 (*CSF3R*) in aCML has prompted the consideration of ruxolitinib to halt the aberrant signaling through JAK inhibition [69]. T618I, a specific mutation of *CSF3R*, causes a lethal myeloproliferative disease in mice, and the degree of splenomegaly and leukocytosis has been demonstrated to be reversed by ruxolitinib [70]. In a case report of a patient with mutated *CSF3R*-*T618I*, hydroxyurea-refractory aCML, the use of ruxolitinib led to a reduction in constitutional symptoms, leucocytosis, and the spleen size, as well as an improvement in anemia and thrombocytopenia [71]. Another case study of ruxolitinib in an 11-year-old patient with aCML resulted in a reduction in leukocytosis and served as a bridge to HSCT [72]. Dao et al. included 23 aCML patients in a phase II trial of ruxolitinib in aCML and CNL patients [73]. Six patients with aCML were found to have the *CSF3R* mutation; two patients were non-responders, while four patients reached the end of cycle six of ruxolitinib treatment [73]. In addition, two patients with aCML had PRs [73]. It is recommended that JAK inhibition with ruxolitinib is used as a bridge to HSCT in aCML patients, especially those with *CSF3R T618I* or *JAK2V617F* mutations [48,74].

#### 3.3.3. Venetoclax

Venetoclax is a BCL-2 inhibitor used in chronic lymphocytic leukemia (CLL), small lymphocytic lymphoma (SLL), and AML patients who are not eligible for standard chemotherapy. Montalban-Bravo et al. retrospectively investigated the activity of venetoclax-based therapy in 27 CMML patients and 26 patients with AML-MRC with anteceding CMML [75]. Ventoclax was given in combination with HMA in 70–60% of patients with CMML. The ORR among CMML patients was 67% with a CR of 4%, a marrow CR with a hematologic improvement of 11%, and a marrow CR of 48% with an overall median duration response of 4 months [75]. All treatment-naïve patients achieved a response, with 28% of patients bridged to allo-HSCT [75]. Interestingly, BCL2 expression was not associated with a therapeutic response [75]. Clinical trials using venetoclax, including NCT04550442, are actively enrolling patients with CMML.

#### 3.3.4. Immune-Modulatory Agents (IMiDs)

Lenalidomide and thalidomide are immune-modulatory agents with several mechanisms of action, including antiangiogenic properties, the repression of IL-6 production, and the activation of apoptotic pathways [76]. Lenalidomide is highly effective in the treatment of multiple myeloma as well as mantle cell lymphoma, chronic lymphocytic leukemia, and some MDS subtypes and has been investigated in MDS trials that included CMML patients [77,78,79]. In a phase I study of 20 CMML patients, PR was achieved in one patient and stable disease (SD) was achieved in nine patients, with a median OS of 28.9 months without significant toxicities [80]. Buckstein et al. studied low-dose melphalan and lenalidomide as possible antiangiogenic therapies in CMML (n = 12) and higher-risk MDS (n = 8) patients [81]. In the 19 patients evaluated, three total responses were seen, all in CMML-1 patients [81]. The ORR for CMML was reported as 25%, with 33% in MP-CMML [81]. Overall, severe thrombocytopenia and neutropenia were associated with therapy, along with other non-hematological toxicities [81]. Sekeres et al. conducted a phase II/III trial investigating AZA combined with lenalidomide or vorinostat (analyzed in the below sections) in 277 patients with higher-risk MDS, including 53 patients with CMML [82]. Patients with CMML treated with AZA + lenalidomide had an ORR of 68% compared to 28% with AZA alone; however, the remission period and OS were similar [82]. The median response duration for all patients was 19 months [82]. Kenealy et al. enrolled 160 patients with MDS and AML, including 22 patients with CMML, investigating lenalidomide in combination with AZA [83]. Overall there was no difference with regard to the clinical benefit (65% in AZA vs. 54% in lenalidomide + AZA, *p* = 0.2) as well as the ORR (57% in AZA vs. 69% in lenalidomide + AZA, *p* = 0.14) at 12 months for the entire cohort [83]. The CMML subgroup did not demonstrate improved responses to AZA with the addition of lenalidomide [83].

Lenalidomide has also been used in patients with MDS/MPN-RS-T [84]. Based on a report of two patients with MDS/MPN-RS-T treated with lenalidomide, one patient became transfusion-independent, and one attained complete molecular remission [85]. Other case reports of lenalidomide in patients with MDS/MPN-RS-T demonstrate some clinical response (spleen reduction and/or transfusion independence), albeit with significant side effects [86,87]. In a retrospective analysis of 33 patients with MDS/MPN-RS-T, 12 patients received lenalidomide, showing a hematological improvement rate of 50% with a median duration of lenalidomide treatment of 10.3 months [55]. In a retrospective analysis of 167 patients with MDS/MPN-RS-T, 47 patients (28%) received lenalidomide, with a hematological improvement rate of 53% and a median treatment duration of 11 months [56]. Naqvi et al. found that three out of seven patients with MDS/MPN-RS-T treated with lenalidomide responded to treatment (ORR 42%); two patients achieved transfusion independence; and one patient had improvement in blood cell counts [88]. Among the patients without hematological improvement, three patients had SD, and one patient stopped after two cycles due to dermatologic toxicity [88].

#### 3.3.5. PARP Inhibitors

PARP inhibitors like veliparib are FDA-approved breast and ovarian cancer treatments and have demonstrated preclinical efficacy in primary MPN samples [89]. PARP enzymes repair single-strand DNA break, and their inhibition results in cellular apoptosis [90]. Neoplasms with mutations in DNA damage repair genes such as *BRCA1* and *BRCA2*, which prevent effective homologous recombination, can accumulate mutations, leading to reliance on PARP1 for DNA repair and survival [90,91]. Pratz et al. demonstrated that 40% of primary MPN samples had impaired homologous repair pathways and, thus, increased sensitivity to veliparib [89]. This preclinical evidence indicates that PARP inhibition could impair homologous repair, which can be potentiated by other anti-neoplastic drugs used in CMML. Veliparib enhanced the cytotoxicity of the DNA-damaging agents topotecan and carboplatin in a phase I study including 22 patients with high-risk CMML or non-CML MPN [92]. A total of 64% of patients with high-risk MPN or CMML (14/22 patients) exhibited a clinical response, and 11 of 14 successfully bridged to allo-BMT [92]. Patients with high-risk MPN, CMML, or AML had a median OS of 13.3 months, while MPN and CMML patients with an extended duration of PARP inhibition had an ORR of 67% and an OS of 15.8 months, without significant adverse events [92]. Six patients with relapsed or refractory AML stemming from CMML were included in a phase I study investigating veliparib in combination with temozolomide; one patient had a CR and two patients had SD with hematological reduction, the stabilization of WBC, and the clearance of circulating blasts [93].

#### 3.3.6. RAS Pathway Inhibition

Trametinib is a mitogen-activated protein kinase 1 MEK1/MEK2 inhibitor that inhibits ERK phosphorylation in the RAS pathway and has demonstrated a reduction in NRAS-mutated AML proliferation in pre-clinical studies [94]. Approximately 70% of MPN-CMML patients have RAS mutations which may contribute to the CMML transformation to AML [20]. Borthakur et al. investigated trametinib in a study including 11 patients with relapsed or refractory CMML [95]. The CMML cohort had the highest ORR (27%) in comparison to the AML and MDS cohorts, with 3 of 11 CMML patients responding to treatment [95]. Two CMML patients were included in a phase I study of salirasib, an oral RAS inhibitor that dislodges RAS molecules from their membranes [96]. One of two patients with CMML demonstrated improvements in platelet counts from 12 × 10^9^ to 110 × 10^9^ for 22 weeks [96]. A randomized controlled trial of 299 patients with high-risk myelodysplastic syndromes after the failure of AZA or DAC, which included 11 CMML patients, investigated rigosertib, a synthetic benzyl sulfone that binds to the Ras-binding domain of various intracellular proteins, including RAF and PI3K [97,98]. No difference in the median OS was reported between the two groups (8.2 months in the rigosertib vs. 5.9 months in the best supportive care group) [98]. Navada et al. included one CMML patient in a phase I/II study of rigosertib in combination with AZA in patients with MDS or AML. The CMML patient had complex cytogenetics and prior HMA therapy and was treated for 19 weeks with the best IWG response of SD [99]. In a case report of aCML, Khanna et al. reported an 81-year-old patient with NRAS-G12D-positive aCML who experienced a rapid reduction in WBC counts, immature granulocytes, and an increase in platelets after trametinib, which aligned with a near complete response for over 14 months [100]. RAS pathway inhibition presents a promising therapeutic option which will require future study to identify mechanisms of resistance as well as its potential for increased efficacy in combination therapy.

#### 3.3.7. Histone Deacetylases Inhibitors

Histone deacetylases inhibitors (HDACIs), including panobinostat (PAN), entinostat, and vorinostat, can lead to an increased expression of genes, inducing apoptosis, cellular differentiation, and cell-cycle arrest, that are aberrantly suppressed in oncogenesis [101]. HDACIs have shown promising preclinical and clinical results in MDS and AML [102,103,104]. Kobayashi et al. investigated PAN and AZA in MDS and CMML patients. Four CMML patients were enrolled; SD was reported as the best response, with tolerable side effects [105]. A phase Ib/IIb clinical trial of PAN and AZA included 4 CMML patients in Ib and 13 CMML patients in the IIb arm [106]. The MDS/CMML patients had a CR of 29.0% in the PAN+AZA arm compared to 10.3% in the AZA arm, with a similar safety profile [106]. However, no significant improvement in OS or time to progression was reported [106].

In contrast, a phase II randomized trial (n = 149, including five patients with CMML) investigated entinostat with AZA, which resulted in a median OS of 22.2 months for treatment with AZA alone as opposed to 14.7 months with AZA and entinostat [107]. The combination of AZA and entinostat led to decreased demethylation compared to that observed in the AZA monotherapy arm, suggesting an antagonistic therapeutic effect [107]. As mentioned in the lenalidomide section, the study of Sekeres et al. showed no significant differences in ORR in CMML patients treated with AZA and vorinostat compared to AZA alone [82].

#### 3.3.8. Other Therapies

Other targeted therapies have been evaluated in a few clinical studies of MDS/MPN—mainly, CMML. Lenzilumab is a monoclonal antibody against GM-CSF, which induces apoptosis in cells with high GM-CSF receptor expression, which is a protein often found on myeloid progenitor cells [65,108]. As mentioned, 90% of CMML patient samples have demonstrated increased proliferation and dependent phospho-STAT5 signaling induced from GM-CSF [65]. When treated with the anti-GM-CSF antibody, CMML BM cells demonstrated apoptosis, particularly in samples with *Ras/CBL/KAK2* signaling mutations [65]. A phase I clinical trial of lenzilumab in patients with CMML showed a durable clinical benefit in 33% of treated patients, without grade III or IV adverse events, with one patient qualifying for allo-HSCT [108]. CD123, the α chain of the IL3 receptor, is highly expressed in CMML progenitor cells and can be targeted by tagraxofusp, a truncated form of the diphtheria toxin with IL-3 [109]. Preliminary data from 36 patients with CMML treated with tagraxofusp showed a marrow CR in 11% of patients, 42% of patients achieved a >50% reduction in spleen size, and one patient was bridged to allo-SCT [110].

Pevonedistat is a small molecule inhibitor of the neural precursor cell (NEDD8)-activating enzyme leading to downstream protein ubiquitination [111]. Pevonedistat treatment has led to AML regression in murine models and has demonstrated a 50% complete response rate (CRR) in treatment-naïve AML patients [112,113]. A randomized phase II trial evaluated pevonedistat in high-risk MDS/CMML or low-blast AML. A subgroup analysis of CMML patients (n = 17) reported a median OS of 21.7 months and an EFS of 21 months for pevonedistat with AZA (in the AZA monotherapy arm, the OS and EFS could not be evaluated) [47]. The ORR was 77.8% for pevonedistat and AZA compared to 75% in the AZA arm alone within the CMML subgroup analysis [47]. In *PANTHER*, a randomized phase III clinical trial of pevonedistat and AZA vs. AZA monotherapy, 27 CMML patients demonstrated a similar OS and EFS between arms, with an ORR of 44% in the pevonedistat plus AZA arm compared to 36% in the AZA monotherapy arm [114].

Omacetaxine mepesuccinate (OM) is a semi-synthetic form of hemoharringtonine (HHT), a plant alkaloid with historical significance as an antineoplastic therapy in China [115]. HHT prevents protein synthesis elongation via interaction with the ribosomal A site and consequentially induces the creation of proteins with a short half-life [115]. Studies of relapsed/refractory AML and MDS show that 16–25% CR rates have been documented, albeit with adverse side effects, including hypotension, diarrhea, and tumor lysis syndrome [116]. OM has FDA approval for chronic or accelerated phase CML resistant to two or more tyrosine kinase inhibitors [117,118]. Short et al. conducted a study of OM in 33 MDS and 8 CMML patients who had previously failed or were intolerant to HMAs, finding an ORR of 33% and a significantly longer survival time and one-year OS rate in OM responders (median OS 8.2 and 41% one-year OS in OM responders vs. 6.3 months and 16% in non-responders) [119]. No difference in response between CMML and MDS patients was observed in a post-hoc analysis [119]. Among CMML patients, one of eight had a marrow CR, and one of eight had hematological improvement [119]. Daver et al. included one patient with CMML in a phase II study of IV OM, who achieved a reduction in bone marrow blasts from 18% to 8% after two courses, without IWG criteria for hematologic improvement [120].

PEG-IFN-alpha was utilized in CML before imatinib development and may have some limited utility in MDS/MPN, including in aCML and MDS/MPN-RS-T. In a retrospective analysis of aCML patients, only one of seven patients treated with IFN-alpha responded (complete hematological response for 100+ months) [121]. In a phase II study of PEG-IFN-α-2b therapy in BCR-ABL-negative myeloproliferative disorders, CR was observed in two of five patients with aCML [122]. Both patients experienced drug toxicities at 36 and 38 months and were taken and then taken off the study [122]. PegIFN-alpha has limited evidence for use in MDS/MPN-RS-T but may be useful in patients needing cytoreductive therapy [84]. PegIFN-alpha treatment should be weighed against the potential to worsen anemia [84].

## 4. Future Directions

The ABNL-MARRO study is an international collaboration that enables a framework for creating clinical trials for the heterogenous MDS/MPN population. The ABNL-MARRO study is currently enrolling patients in a trial investigating itacitinib, a selective JAK1 inhibitor with an oral combination of DAC and the cytidine deaminase inhibitor, cedazuridine, as the first investigation in an international clinical trial [123].

## 5. Allogeneic Transplantation

### 5.1. CMML

The only therapeutic modality that is potentially curative for patients with MDS/MPN is allogeneic bone marrow transplantation (allo-BMT). To date, many studies have explored the clinical outcomes of allo-BMT for patients with MDS/MPN. Most studies have evaluated a small subpopulation of CMML, in conjunction with a larger pool of MDS or AML patients and, to a lesser extent, by itself. One of the first retrospective studies evaluating allo-BMT outcomes in 21 patients with CMML was performed by Zang et al. in the Fred Hutchinson Cancer Research Center, reporting a 3-year disease-free survival (DFS) and relapse rate (RR) of 39% and 25%, respectively [124]. A shorter duration from diagnosis to BMT was associated with more favorable clinical outcomes [124]. Two years later, a European Blood and Marrow Transplantation Registry (EBMT) study evaluated 50 patients with CMML receiving allo-BMT and reported 5-year OS, DFS, and RR values of 21%, 18%, and 49%, respectively but a transplant-related mortality (TRM) of 52% [125]. The incidence of acute GVHD was negatively associated and T cell-depleted allografts were positively associated with relapse, factors that indicate a “graft-versus-CMML” effect [125]. In 2005, Kerbauy et al. investigated 43 patients with CMML, finding that allo-BMT led to a 4-year relapse-free survival (RFS) and cumulative incidences of 41% and 23%, respectively [126]. Patients with a higher hematopoietic cell transplantation-comorbidity index (HCT-CI) exhibited overall worse survival scores [126]. The HCT-CI considers and assigns one score to various comorbidities and has been used as a tool to predict outcomes of HSCT based on the burden of comorbidities of patients [127]. Similarly, Sharma et al., in a study of 35 patients with CMML, found that high HCT-CI (>3) was associated with worse survival outcomes. Additionally, splenomegaly and engraftment failure were predictors of worse OS in patients without and with blast transformation, respectively [128].

Additional clinical studies have evaluated clinical and molecular prognostic factors of allo-BMT in CMML. A French study analyzed outcomes in 73 patients with CMML who received allo-BMT between 1992 and 2002 [129]. Transplants before 2004 and palpable splenomegaly at the time of transplantation were associated with a decreased OS, while a transplant before 2004 was also associated with higher non-relapse mortality (NRM) [129]. A UK study from Krishnamurthy et al. investigated 18 patients with CMML who received allo-BMT, finding the 3-year OS, NRM, and relapse incidence to be 31%, 31%, and 47%, respectively. A better DFS was observed in patients with favorable cytogenetics and those with BM blasts < 5% [130]. An analysis from Japan investigated the effect of donor selection in 159 patients with CMML. An improved OS was noted with HLA-matched related donor BM (50.4%), followed by unrelated BM (31.4%), HLA-mismatched related BM (16.7%), and unrelated cord (UCB) blood (15.4%) [131]. In a multivariate analysis using HLA-matched related BM as a control, UCB was correlated with a higher TRM (HR = 3.32, *p* = 0.01) [131]. A study evaluating the prognostic impact of various genetic and clinical factors and prognostic systems identified that worse outcomes were associated with abnormal karyotype, leukopenia (WBC < 2 × 10^9^/L), and neutropenia (ANC < 1.5 × 10^9^/L) at the time of transplant [132]. Better survival after transplant was observed in patients with myelodysplastic, as opposed to myeloproliferative, CMML [132]. Despite the negative impact of the abnormal karyotype, none of the most prevalent *ASXL1*, *SRSF2,* and *TET2* mutations had a prognostic impact [132]. Gagelman et al. evaluated 240 patients with CMML with a median follow-up of 5.5 years and proposed a prognostic system (named *CMML transplant score*) that accounts for *ASXL1* and/or *NRAS* mutations (four points), a percentage of BM blasts > 2% (four points), and a comorbidity index (one point for eight different comorbidities), which cumulates in five risk groups with significant differences in survival and non-relapse mortality [133].

A European Group for Blood and Marrow Transplantation study retrospectively evaluated transplantation outcomes in 513 patients with CMML and reported 4-year NRM, RR, DFS, and OS values of 41%, 32%, 27%, and 33%, respectively [134]. The only factor associated with both better RFS and OS in this study was the presence of CR at the time of transplant [134]. Predictors of better RFS alone were good-risk cytogenetics, a low HCT-CI, a high pretransplant hematocrit, and a lower age [134]. Although grades three to four acute GVHD occurred in 21 patients (26%) and chronic GVHD occurred in 37 patients (44%) at two years, only 2 patients died due to GVHD [134]. The presence of blast transformation in MDS/MPN, as expected, has been shown to dramatically decrease the probability of survival post-transplant, with a 3-year OS as low as 18% [135]. A longer follow-up of CMML patients was performed by Eissa et al., who followed up 85 patients up to 19 years post-transplant, reporting 10-year NRM, RR, and PFS values of 34%, 27%, and 38% [136]. Predictors of worse PFS were increasing age, higher HCT-CI, lower pre-HTC hematocrit, and high-risk cytogenetics [136].

Another multicenter study from Liu et al., which utilized data from the Center for International Blood and Marrow Transplant Research (CIBMTR), investigated prognostic factors associated with transplant outcomes in 209 adult patients with CMML receiving allo-BMT [137]. A lower performance status, bone marrow graft, and higher CMML-specific prognostic scoring system (CPSS) scores were associated with worse survival outcomes, particularly after relapse [137]. Different conditioning regimens (MAC vs. RIC) displayed no differences in outcomes [137]. CPSS considers factors such as CMML FAB type, CMML WHO type, CMML-specific cytogenetics, and RBC transfusion dependence. It has traditionally been used in the non-transplant setting. The above study established the utility in the prognostication of allo-BMT for CMML [138]. Another study from Gagelmann et al. found survival differences based on the stratification of patients based on CPSS scores in lower-risk (low/intermediate-1 CPSS score) and higher-risk (intermediate-2/high CPSS score) groups, with higher-risk patients benefiting more in the setting of allo-BMT [139]. A retrospective study by Robin et al. evaluated 1114 patients with CMML (diagnosed between 2000 and 2014) [140]. It concluded that allo-BMT in patients with a lower risk was associated with decreased survival (5-year OS 20% with allo-HSCT vs. 42% without allo-HSCT) [140]. In comparison, increased survival was observed for intermediate-2/high CPSS scores (higher risk) [140]. In both risk groups, allo-BMT significantly increased the mortality risk for the first two years after the transplant [140]. While the CPSS score can be utilized as a prognostic marker, a system utilizing both clinical and molecular features is more useful. A group from Fred Hutchinson evaluated 129 patients with CMML and reported a 10-year PFS of 29% [141]. Adverse cytogenetics, a high CPSS score, a high mutational burden (≥10 mutations), and ≥four mutated epigenetic regulatory genes were associated with an increased risk of relapse, while adverse cytogenetics and an HCT-CI score ≥ four was associated with increased mortality [141].

Mortality after transplant is mainly caused by transplant-related mortality (TRM) or the relapse and progression of disease. The TRM in the first 100 days post-transplant is primarily driven by severe acute GVHD, with different reports finding this percentage to range from 25 to 41% [142]. Options for patients that relapse post-transplant are limited, and survival outcomes are poor. Donor lymphocyte infusion (DLI) involves the infusion of unmanipulated lymphocytes from the donor of the allo-BMT, used as an immunotherapy to trigger GvL in patients with myeloid malignancies that relapse post-allo-BMT [143]. DLI in CMML patients who relapsed post-transplant led to CR in three out of seven patients, but at the expense of an overall high transplant-related mortality (41%) [144].

### 5.2. Studies of aCML, MDS-MPN-NOS, and MDS/MPN Combinations

The utilization of allo-BMT for aCML was first described in a case report [145]. In 2004, the first clinical trials of allo-BMT for aCML were performed by Koldehoff et al. and Mittal et al., which demonstrated complete remission in nine out of nine aCML and six out of seven aCML (and three out of eight CMML patients) patients in each respective study [146,147]. There have been few studies focused exclusively on aCML. Itonaga et al. retrospectively followed 14 aCML patients and underlined the importance of an effective reduction in BM blasts before allo-BMT, as the presence of blasts < 5% was associated with a four-times-increased 1-year OS (76 vs. 20%) [148]. The most extensive retrospective study of aCML patients utilized data from the European Society for Blood and Marrow Transplantation (EBMT) registry and included 42 patients, reporting a 5-year OS, RFS, NRM, and RR of 51%, 36%, 24%, and 40%, respectively [149]. A younger age and a lower EBMT risk score were associated with overall more favorable survival outcomes [149]. EBMT risk is a tool that calculates the pre-transplant risk, considering factors like the recipient’s age, donor’s type, donor–recipient gender combination, stage of disease, and time from diagnosis [150].

Few studies have exclusively evaluated the clinical outcomes of allo-BMT in MDS/MPN-NOS. A collaborative analysis between the Mayo Clinic and Moffitt investigated outcomes in MDS-MPN-NOS patients, identifying 63% who remained alive and disease-free at a median follow-up time of 61 months [53]. A Japanese study with 86 MDS-MPN-NOS patients revealed a 3-year OS, NRM, and RR of 48.5%, 26.3%, and 23.7%, respectively [151]. Finally, a Mayo Clinic study of eight patients with MDS-MPN-NOS showed OS and NRM values of 62% and 14% in a median follow-up of 15 months [128].

Despite the uniqueness of each MDS/MPN entity, studies have reported combined outcomes, often because primary analysis shows no differences after stratifying for each neoplasm. A recent collaborative analysis of 15 North American centers evaluated the haploidentical BMT with PTCy in 120 patients with MDS/MPN and reported a 3-year NRM, PFS, relapse, and OS, of 25%, 48%, 27%, and 56%, respectively [152]. Severe (grade III–IV) acute GVHD and severe chronic GVHD were manifested in only 12% and 14% of patients, respectively, highlighting the utility of PTCy to reduce the incidence of GVHD and improve the post-BMT outcomes in these patients [152]. A strong association between survival outcomes was identified with increasing age, splenomegaly, and mutations in *EZH2/RUNX1/SETBP1* genes [152]. A mutational analysis of genes including *ASXL1*, *CBL*, *TET2*, and *NRAS* used next-generation sequencing as a predictor for relapse after transplant in MDS/MPN [153]. Patients with a detectable mutation pre-and post-transplant had a higher incidence of relapse (50%) than those in which the mutation was only detected before the transplant but not after (15%) [153]. These molecular markers, the *CSF3R* T618I mutation, and the detection of MRD with PCR for WT1 or flow cytometry can be used as predictors of relapse post-transplant [154,155].

### 5.3. Preparation Regimens and Bridging Therapy

Another area of research lies in the conditioning regimens and bridging therapies before allo-HSCT. Traditionally, myeloablative conditioning (MAC) involving cytotoxic agents, like busulfan with cyclophosphamide and/or total body irradiation (TBI) ≥ 5 Gy, is preferred in young patients. In contrast, reduced-intensity conditioning (RIC) regimens are usually preferred in older patients or patients with comorbidities. A study comparing MAC and RIC in patients with MDS/MPN syndromes showed relapse in all patients (5/10) receiving RIC but no relapse in those pre-treated with MAC (5/10), underscoring the survival benefit associated with more intense myeloablation [156]. However, the majority of patients with MDS/MPN are older, potentially with comorbidities, which are factors that limit their eligibility for MAC. The choice of the specific MAC can also be important. An intermediate total body irradiation (TBI) of 6–8 Gy with fludarabine was associated with improved RR (but not NRM) compared to alkylator-based conditioning (fludarabine with treosulfan or busulfan) [156]. A study from Wedge et al. showed that myeloablative conditioning with fludarabine and treosulfan in an MDS and CMML cohort resulted in a better one-year OS (84%), as opposed to standard MAC (58.3%) and non-MAC (68.3%) regimens [157]. TBI has been evaluated in additional studies of CMML with MDS, reporting that increasing doses to 450cGy can achieve lower rates of HSCT failure [158]. Finally, myeloablation with total lymphoid irradiation (TLI) and anti-thymocyte globulin (ATG) have been evaluated, showing promising results, with an overall incidence of aGVHD II-IV of 14% and a 36-month NRM of 11% in a mixed cohort of MDS, MPN, and CMML patients [159]. Except for in preparative regimens, bridging chemotherapy before transplant can affect survival outcomes. Treatment with an HMA prior to allo-BMT has been associated with a significantly lower 3-year cumulative incidence of relapse (22%) and a higher PFS (43%) as opposed to treatment with cytotoxic chemotherapy (35% and 27%, respectively) [160]. Kongtim et al. concluded that induction with an HMA can facilitate the achievement of remission and increase PFS [160].

**Figure 1:**
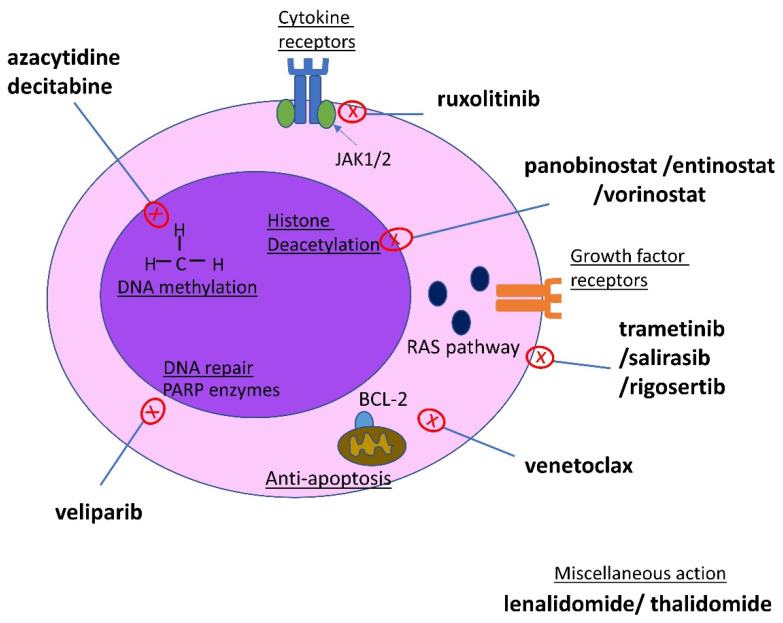
Schematic of therapies most commonly used in MDS/MPN syndromes that target specific pathways in a malignant myeloid cell.

Table 1 summarizes the currently available therapeutic strategies for MDS/MPN neoplasms, and Table 2 summarizes agents that have been evaluated in early-phase clinical trials. Figure 1 summarizes the mechanisms of the currently available therapies in MDS/MPN neoplasms.

## 6. Conclusions

MDS/MPN overlap syndromes represent a unique and challenging group of hematological disorders. The understanding of these diseases has significantly advanced, particularly regarding their underlying biology and genetic abnormalities, providing insights into the pathogenesis and potential therapeutic targets. Current treatment approaches encompass a multimodal strategy, including disease-modifying agents, targeted therapies, and supportive care, with allo-BMT being the only potentially curative option for eligible patients. However, challenges remain in optimizing patient selection, refining transplant techniques, and minimizing transplant-related complications. Future clinical research directions on MDS/MPN overlap syndromes should focus on several key areas. To guide personalized therapy decisions, efforts should be made to develop risk-adapted treatment algorithms based on comprehensive genetic profiling, disease characteristics, and patient factors. Refining allo-BMT protocols, such as RIC regimens and improved GVHD prophylaxis strategies, holds promise in reducing treatment-related morbidity and mortality. Moreover, identifying reliable biomarkers for disease monitoring, response assessment, and the early detection of relapse would greatly enhance clinical management. Ultimately, collaborative research endeavors and international registries are vital, as they facilitate the accumulation of larger patient cohorts, leading to a better understanding of disease heterogeneity and refining prognostic models.

## Figures and Tables

**Table 1:** Standard-of-Care Treatments in CMML.

Agent	Mechanism of Action	Response	Reference
HMA (Azacitidine and Decitabine)	Irreversibly bind DNA transferase, cause direct DNA damage [34,35,36,37]	ORR: 17–75% CR: 7–45%	[38,39,40,41,42,43,44,45,46,47]
Ruxolitinib	JAK1/2 Inhibitor	ORR: 0–38%	[66,67,68]
Lenalidomide	Immune-modulatory agent, anti-angiogenic properties, cytokine repression, activation apoptotic pathways [76]	ORR: 25–69%	[80,81,82,83]
Venetoclax	BCL-2 inhibitor	ORR: 67%CR: 4%	[75]
BMT	Infusion of bone marrow stem cells post-preparation regimen	3 yr DFS: 39%; RR: 25% [124] 3 yr OS, NRM, Relapse incidence: 31%, 31%, 47% [130] 4 yr NRM, RR, DFS, and OS: 41%, 32%, 27%, and 33% [134] RFS 4 yr: 41% [126] 5 yr DFS: 18%, RR: 49% [125] 10 yr NRM, RR, PFS: 34%, 27%, and 29–38% [136,141]	[124,125,126,128,130,134,135,136,137,139,140,141]

**Table 2:** Agents that have been evaluated in MDS/MPN patients in clinical trials.

Agent	Target/Mechanism	Disease	Type of Study	Response	Other Outcomes	Reference
Veliparib + topotecan and carboplatin	PARP inhibition + chemotherapy	High-risk MPN, CMML, AML	Phase I	ORR 67%	Median OS 15.8 months	[92]
Veliparib + temozolomide	PARP inhibition + chemotherapy	AML arising from CMML	Phase I	One patient had CR, two patients had SD	Two patients had counts normalization and clearance of circulating blasts	[93]
Trametinib	MEK1/MEK2 inhibition	Relapsed/Refractory CMML	Phase I/II	ORR 27%		[95]
Salirasib	RAS inhibitor	CMML	Phase I		One of two CMML patients showed platelets improvement	[96]
Rigosertib	RAS inhibition	CMML	Phase III	ORR 0%, SD 35%	No difference in OS compared to supportive care	[98]
Rigosertib + Azacitidine	RAS inhibition + HMA	1 CMML patient	Phase I/II	SD		[99]
Panobinostat + Azacitidine	HDAC inhibition + HMA	4 CMML patients	Phase I	SD 100%		[105]
Panobinostat + Azacitidine	HDAC inhibition + HMA	17 CMML patients	Phase Ib/IIb	CR 29% compared to 10.3% with azacitidine alone	Similar safety profile	[106]
Entinostat + Azacitidine	HDAC inhibition + HMA	5 CMML patients	Phase II	Response 32% with azacitidine alone compared to 27% with combination	OS 22 months with azacitidine alone compared to 14.7 months with combination, antagonistic effect based on methylation profile	[107]
Lenzilumab	Antibody against GM-CSF	CMML	Phase I	Clinical benefit 33%	No grade III or IV adverse events	[108]
Tagraxofusp	Anti-CD123 drug conjugate	Relapsed/Refractory CMML	Phase I/II	CR 11%	Splenic reduction 42%	[110]
Pevonedistat + Azacitidine	NEDD88 inhibitor + HMA	17 CMML patients	Phase II	ORR 77.5% with combination compared to 75% with azacitidine alone		[47]
Pevonedistat + Azacitidine	NEDD88 inhibitor + HMA	27 CMML patients	Phase III	ORR 44% with the combination compared to 35% with azacitidine alone	Similar OS and EFS between arms	[114]
Omacetaxine mepesuccinate	Semi-synthetic form of HHT, plant alkaloid preventing protein synthesis	8 CMML patients, progressed on HMA	Phase II	ORR 33%	Median OS 7.5 months, grade III adverse events 26%	[119]
PEG-IFN-alpha	Inhibition of protein synthesis and cytotoxicity	5 aCML patients	Phase II	CR 40%	Significant toxicity	[122]

ORR overall response rate, CR complete remission, SD stable disease, OS overall survival, EFS event-free survival.
